# The relationship between least-cost and resistance distance

**DOI:** 10.1371/journal.pone.0174212

**Published:** 2017-03-28

**Authors:** Robby R. Marrotte, Jeff Bowman

**Affiliations:** 1 Environmental & Life Sciences Graduate Program, Trent University, Peterborough, Ontario, Canada; 2 Wildlife Research & Monitoring Section, Ontario Ministry of Natural Resources and Forestry, Peterborough, Ontario, Canada; Nankai University, CHINA

## Abstract

Least-cost modelling and circuit theory are common analogs used in ecology and evolution to model gene flow or animal movement across landscapes. Least-cost modelling estimates the least-cost distance, whereas circuit theory estimates resistance distance. The bias added in choosing one method over the other has not been well documented. We designed an experiment to test whether both methods were linearly related. We also tested the sensitivity of these metrics to variation in Euclidean distance, spatial autocorrelation, the number of pixels representing the landscape, and data aggregation. We found that least-cost and resistance distance were not linearly related unless a transformation was applied. Resistance distance was less sensitive to the number of pixels representing a landscape and was also less sensitive than least-cost distance to the Euclidean distance between nodes. Spatial autocorrelation did not affect either method or the relationship between methods. Resistance distance was more sensitive to aggregation in any form compared to least-cost distance. Therefore, the metric used to infer movement or gene flow and the manipulations applied to the data used to calculate these metrics may govern findings.

## Introduction

**Fig 1:**
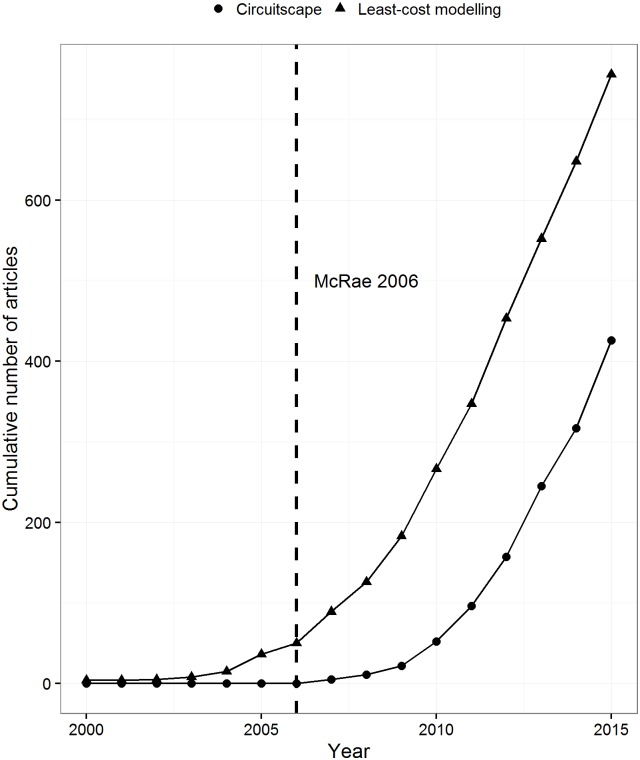
The use of least-cost modelling and circuit theory in ecology. Cumulative increase in the number of Google Scholar articles that have mentioned Circuitscape and least-cost modelling in the field of Ecology since 2000.

Early work by Doyle and Snell [1] revealed that current in an electrical circuit travels similarly to a random walk. Two decades later McRae [2] applied this concept to model gene flow. Since then, circuit theory and associated software Circuitscape [2], has been used to simulate movement and gene flow of a multitude of species in the fields of ecology and evolution (e.g., [3], [4], [5], [6], [7]). At the same time, least-cost modelling has also been commonly used for very similar applications (e.g., [8], [9], [10], [11], [12]). The popularity of these methods in ecology is quickly increasing (Fig 1). A Google Scholar search with the query: “least-cost modelling” AND “ecology”, revealed that peer reviewed articles mentioning this approach have increased from 4 articles in 2000 to 108 in 2015. On the other hand, the search query: “Circuitscape” AND “Ecology”, revealed 0 articles in 2000 and 109 articles in 2015. Circuitscape was first conceived by McRae [2], however the name of the software was not coined in peer reviewed literature until 2007 [7], [13], [14]. Conversely, least-cost modelling in ecology has been around since the early 2000s [15], [16], [17]. While the usage of both approaches is likely to increase, few studies have investigated their quantitative differences [18], [19], [20].

Both methods have the common objective of predicting gene flow or movement between locations while considering the influence of the landscape. In both cases, the landscape is represented as a cost surface, where a high cost is considered highly resistant to movement. Consequently, the distance or time to travel between locations is hindered by the cost to travel through obstacles. The straight Euclidean path is deviated around obstructions causing spatial distortion that is not in accordance with Tobler’s 1^st^ law of geography [21], [22]. Both continuous and discrete (categorical) resistance surfaces are frequently used to infer movement and gene flow of populations or individuals. Continuous resistance surfaces are frequently used to infer the effect of gradients of a continuous variable such as elevation [23], [24], snow depth [25], temperature [26], slope [27], habitat suitability [28], or species distribution [29] on the movement pattern or gene flow of a species. On the other hand, discrete resistance surfaces are often generated from land use [30] or land cover maps [31], [32].

The relationship between least-cost and resistance distance has not been studied in great depth [18], [19], [20], and researchers ought to recognise the bias they might be adding when choosing one method over another. Both methods are different in the way movement is characterised. Least-cost modelling expresses movement as the least-cost path between two focal points. Circuit theory expresses movement as the probability of a random walk between focal nodes [33]. The distance between nodes is then expressed as the accumulated cost of the least-cost path or the resistance distance [15], [34]. However, both distance measures are similar in accounting for the influence of the landscape on the movement or gene flow of individuals. Both measures also complement one another and have been used together for this very reason (e.g., [35]). Furthermore, there is an apparent relationship between the methods. In a scenario where only one pathway exists between two nodes, the resistance distance is equal to the least-cost distance [33]. Where there are multiple independent pathways between nodes, the average least-cost distance of these pathways is equivalent to the resistance distance. Therefore, a coarse measure of the number of pathways or the redundancy between nodes is the ratio between least-cost and resistance distance [33]:

$$Redundancy=\frac{\left(least-cost\;distance\right)}{\hat{R}}$$ (1)

where $\hat{\text{R}}$ is resistance distance. Least-cost and resistance methods have been combined in a diverse number of different ways [35], [36], [37], [38]. However, the use of the redundancy metric is rare in the literature [18].

Recently, McClure *et al*. [20] compared the predictability of cost and resistance distance and found that least-cost distance predicted elk migration path slightly better and resistance distance predicted wolverine dispersal paths better. Also, these methods produced different results when used to find habitat patches that should be prioritized for conservation [19]. The importance of patches as connectors to facilitate dispersal was overestimated using least-cost compared to resistance distance for short or medium dispersals. Koen *et al*. [18] showed that if high quality elements of habitat were held constant at low movement cost while low quality habitat was sequentially increased, there was a positive linear change in both least-cost and resistance distance. All other responses were asymptotic for both least-cost and resistance distance. This study highlights the sensitivity of both resistance and least-cost distance to the cost weights assigned to landscape elements. Schwartz *et al*. [6] found that wolverine gene flow was more correlated to least-cost distance compared to resistance distance and both methods only converged in narrow areas. They suggested that least-cost distance may explain southern wolverine gene flow better because these populations are often found in linear habitat connected by chains of mountains. Essentially, the wolverine is restricted within this habitat and its movement behaviour and consequently its gene flow mirrors a least-cost path.

Such findings highlight the importance of investigating assumptions of both effective distance measures. Researchers should choose a movement or gene flow metric that closely matches the behavior, biology, and ecology of the focal entity. For instance, least-cost distance inherently assumes that an animal will follow the path of least resistance between focal locations [6] and resistance distance assumes multiple pathways between sites [2]. We suspect that the data used to quantify these distances are a major contributor that governs how both methods perform. In this study, we define least-cost distance as the cost-distance of the least-cost path between focal points [34]. This metric is often named the accumulated cost and is occasionally confused with the least-cost path length. It is important to recognize the difference, because this latter metric is highly correlated with Euclidean distance and consequently is a poor measure of connectivity [34], unless there is high uncertainty about cost weights [18].

We sought to better understand the difference between these commonly used methods to estimate effective distance between locations by comparing their outcomes on landscapes simulated using spatially correlated random fields. Our prior understanding was that these methods were related by a quantity referred to as redundancy (Eq 1). In addition, we also thought that the number of pathways between focal points monotonically decreases with the Euclidean distance and spatial heterogeneity. Our reasoning was that Euclidean distance and heterogeneity limit movement by reducing the number of pathways between focal areas. Thus, increasing the distance between focal points and the degree of spatial autocorrelation should lead to a divergence between least-cost and resistance distance. We also thought that the relationship between methods could be influenced by the number of pixels representing the landscape. Our logic was that the number of pathways between nodes increases monotonically with the number of pixels. Therefore, as the number of pixels increases we should also see an equivalent increase in redundancy. Finally, we also expected that aggregation in the form of spatial and thematic accuracy should lead to convergence of the two methods. Our reasoning was that aggregation in any form should lead to a reduction in the number of pathways between focal points and subsequently both methods converge with increasing aggregation. Aggregation is very commonly used, since the algorithms using least-cost distance are processor and memory intensive, leading many researchers to spatially aggregate their data at coarse resolutions [6], [39], [40].

## Materials and methods

### Landscape creation

We used unconditional Gaussian simulations [41–43], also known as spatially correlated random fields [44], in the ‘gstat’ package [45] of the R statistical language [46] to generate landscape simulations.

To determine linearity, the effect of Euclidean distance, spatial autocorrelation, and the response of these methods to aggregation we simulated 1,000 landscapes with varying degrees of spatial autocorrelation. We first generated template landscapes with spatial dimensions of 1,000 x 1,000 units; equivalent to 1 million pixels. Next, for each of these landscapes, we created an exponential variogram model with a sill of 0.025 and we assigned a random spatial range. The range of each of these models was randomly sampled from a bounded uniform distribution between 1 and 1000 units. As the range increased from 1 to 1000, so did the degree of spatial autocorrelation between neighbouring pixels. We then predicted the model into Cartesian space as a continuous raster surface, scaled between 1 and 1000 integer values. These values represented landscape resistance or cost of movement, where a high value of 1000 had greater cost of movement. Subsequently, with these same landscapes, we independently performed spatial and thematic aggregation to determine their effect on the congruence between least-cost and resistance distance. We aggregated each landscape by a factor of 1 to 20 to simulate different levels of spatial aggregation. In parallel, we aggregated the cost values into a random number of discrete groups using quantiles to generated different levels of thematic resolution. The number of categories ranged from 2 categories (i.e., a patch-matrix landscape) to 20 possible values (e.g., a land use or land cover classification). Essentially, we used the cost values to generate a ramp of discrete classifications and compared these to the continuous cost landscape.

To determine the effect of the number of pixels on the relationship between least-cost and resistance distance, we simulated an additional 1,000 landscapes with varying number of pixels and degree of spatial autocorrelation. We first assigned a square spatial dimension to each perspective landscape ranging from 100 to 1000 units. This generated square blank landscapes that had from 10,000 to 1 million pixels. As in the previous analysis we modelled this spatial extent with a variogram with a sill of 0.025 and a spatial range randomly sampled for a uniform distribution. We restricted the creation of landscapes that had duplicate representations, that had less than 10,000 pixels, or that had a spatial autocorrelation range larger than the dimension of the spatial extent (e.g., 1000 units).

In both sets of simulations, for each resistance surface generated, we randomly placed 15 focal nodes on the landscape and calculated pairwise measures of least-cost and resistance distance between all node pairs. To reduce edge effect, we did not place sites within a buffer zone within 10% of a landscape’s dimension [47]. To calculate resistance distance, we used Circuitscape [2]. To calculate the least-cost distance we used Dijkstra’s algorithm weighted by cost that is implemented in the cost distance function in the ‘gdistance’ R package [48], [49]. We configured analyses in all platforms to use pairwise modelling with 8 neighbours. We used the average resistance to calculate these effective distances. We also tested average conductance, but found no noticeable differences in our subsequent results.

Opportunistically, we also compared resistance distance to an alternative method that directly estimates the commute-time or the expected time it takes for a random walk from one node to another. Given that commute-time is easily calculated in R, we were interested to evaluate whether commute-time and resistance distance were equivalent, as both reflect the underlying properties of a random walk. To calculate the commute-time, we used the algorithm given by Fouss *et al*. [50] implemented in the commute-time distance function in the ‘gdistance’ R package [48]. We expected a direct, proportional relationship between these distance measures [33], [51].

### Statistical analysis

We compared the effective distance between all pairs for both methods for each landscape represented by 1 million pixels. To determine whether linearity existed between least-cost and resistance distance, and to test the effect of Euclidean distance and spatial autocorrelation on the relationship between least-cost and resistance distance we compared the pairwise distances calculated by each method on each landscape of 1 million pixels with a measure of rank correlation. We did not want to bias our analysis by assuming linearity between both metrics, consequently we used a Spearman’s rank correlation (*p*). In addition, our prior analyses indicated that these measures were not linearly associated (See Results). To determine the effect of the number of pixels representing a landscape on the relationship between these methods we calculated the rate of change of the distances of both measures against the associated number of pixels representing the landscape. We additionally verified the relationship between methods by comparing their distances with a Spearman’s rank correlation on all landscapes represented by 1 million pixels. Finally, to determine the effect of aggregation, we first assessed the concordance of least-cost and resistance distance before aggregation and we then compared this baseline value to those after aggregation. Our measure of change due to aggregation was the difference in the rank correlation (Δ*p*) between the baseline landscape of 1 million pixels and the aggregated landscape for both types of aggregation. We assessed the variability of each method due to aggregation by comparing their measures before and after aggregation with a Spearman’s rank correlation.

## Results

### Linearity between methods

**Fig 2:**
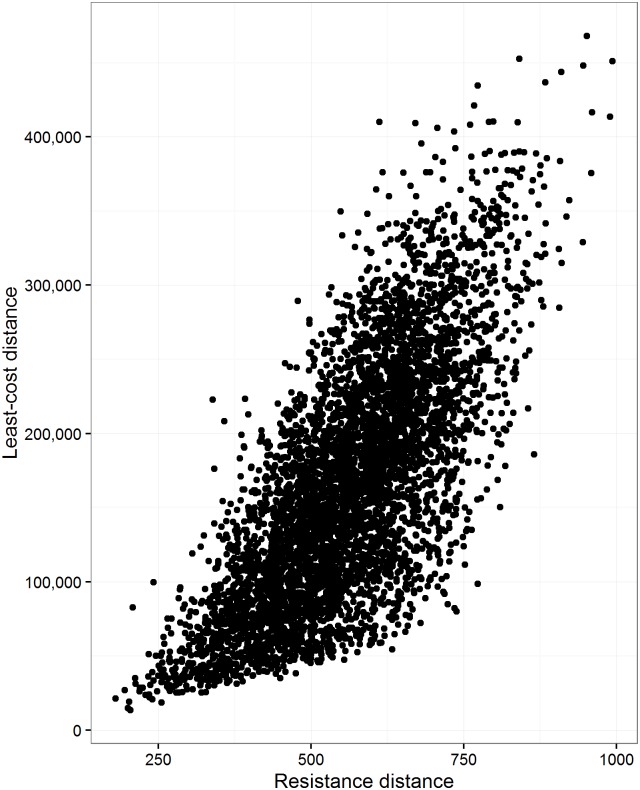
The relationship between least-cost and resistance distance. The relationship between least-cost and resistance distance for a subset of 5,000 random pairs sampled from 1000 different simulated landscapes (*p* = 0.731). We had to randomly subset the data to make the figures more visually appealing, since there were 105,000 actual points. Landscape size (number of pixels) was held constant at 1,000,000.

The relationship between least-cost distance and resistance distance was non-linear (Fig 2). The response was curvilinear and exponential. When we square-root transformed the least-cost distance or squared the resistance distance the response was linearized (S1 and S2 Figs). The distribution of the rank correlation between least-cost and resistance distance for all 1,000 landscapes ranged from 0.25 and 0.93 (μ = 0.720 and σ = 0.101; S3 Fig). The relationship between resistance distance from Circuitscape and commute-time was linear (*p* = 0.87 for a subset of 5,000 paths; S4 Fig). The distribution of the rank correlation between resistance distance and commute-time for all 1,000 landscapes did not vary much from the average (μ = 0.998 and σ = 0.001).

### Euclidean distance and spatial autocorrelation

**Fig 3:**
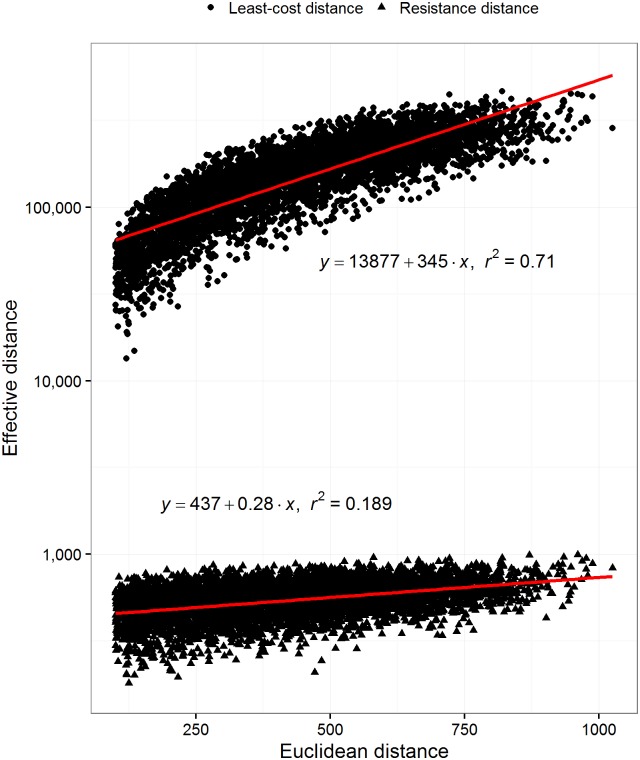
The effect of Euclidean distance. The effect of Euclidean distance on both methods for a subset of 5,000 random paths sampled from 1000 different simulated landscapes represented with 1 million pixels or nodes. The y-axis in this graph is on a logarithmic scale for visualization purposes only. Least-cost distance increases linearly with Euclidean distance while resistance distance increases linearly to quite a lesser extent. Landscape size (number of pixels) is held constant at 1,000,000.

Both least-cost and resistance distance increased monotonically with Euclidean distance (Fig 3). Least-cost distance increased at a much higher rate compared to resistance distance (slope = 345 vs. 0.28). Euclidean distance explained 71.0% of the variation in least-cost distance and only 18.9% of variation in resistance distance. In general, a 100 unit increase in Euclidean space between two focal points led to only a 28 unit increase in resistance distance, compared to a 34,500 unit increase in least-cost distance. The degree of spatial autocorrelation of a landscape did not seem to affect the estimates of either method (S5 Fig) and did not affect their relationship (S6 Fig).

### Number of pixels

**Fig 4:**
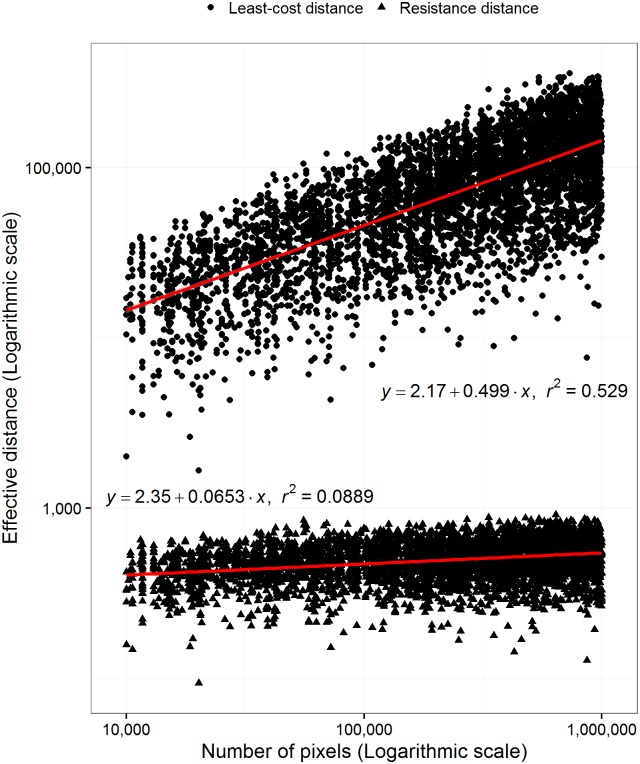
The effect of the number of pixels representing a landscape. The least-cost and resistance distance in relation with the varying number of pixels for a subset of 5,000 random paths sampled from 1,000 deferent landscapes with varying degrees of spatial autocorrelation and number of pixels representing the landscape. Both axes are on a logarithmic scale. In this case both the response and explanatory variables were log-transformed to calculate the linear model. Landscape size (number of pixels) is varied from 10 thousand to 1 million pixels.

**Fig 5:**
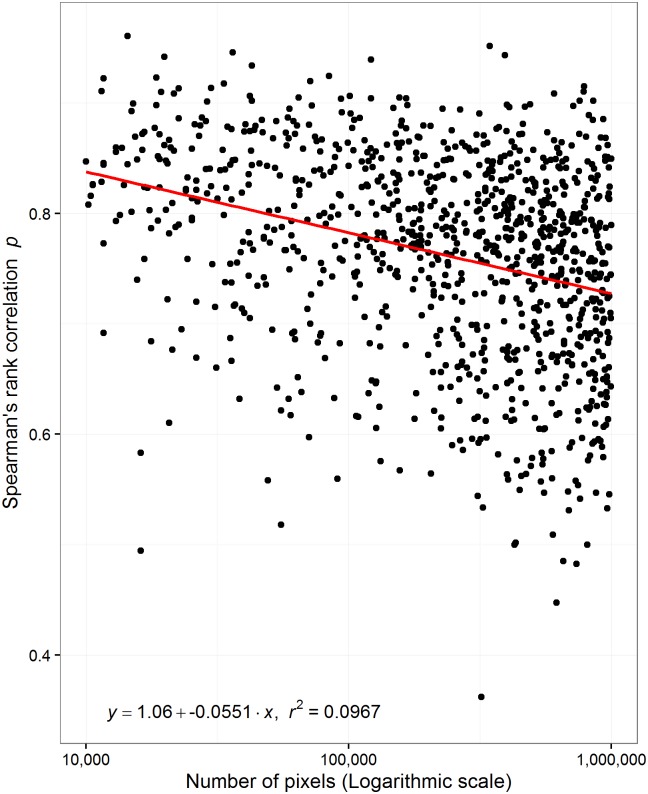
The number of pixels and rank order agreement. The rank agreement (*p*) between least-cost distance in relation with the log transformed number of pixels representing 1,000 different landscapes with varying spatial autocorrelation. The x-axis is on a logarithmic scale. Landscape size (number of pixels) is varied from 10 thousand to 1 million pixels.

For both least-cost and resistance distance there was an exponential response with the number of pixels representing the landscape (Fig 4). In addition, this trend was stronger for least-cost compared to resistance distance. A 100% increase in the number of nodes led to a 50% increase in least-cost distance and only a 7% increase in resistance distance. The trend for least-cost distance was predominantly explained by the number of pixels (r^2^ = 0.53) but this was not the case for resistance distance (r^2^ = 0.09). The Spearman’s rank correlation calculated between both methods between all pairwise combinations of the 15 nodes decreased as the number of pixels representing the landscape increased (Fig 5). This indicated that the agreement between these effective distances decreased as the number of pixels (or the number of pathways) through the landscape increased. For instance, the rank correlation between methods for a landscape represented by 100,000 pixels would be ~0.78, for a landscape represented by 1 million pixels would be approximately 0.73, and a landscape represented by 10 million pixels would be ~0.67. Therefore, we should not expect the same degree of agreement between methods on landscapes represented by different numbers of pixels. In other words, the magnitude of agreement between measures depends on the number of pixels representing the landscape.

### Aggregation

**Fig 6:**
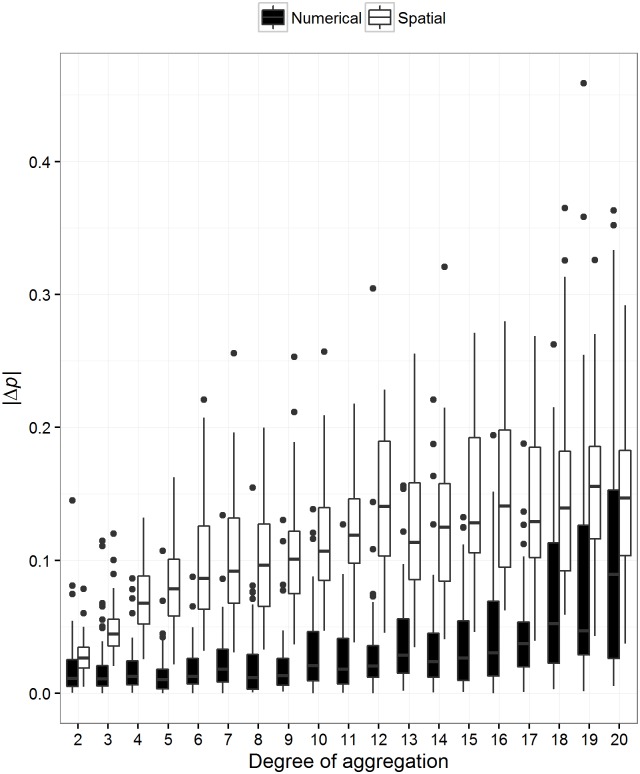
The effect of both spatial and thematic aggregation across methods. The effect of aggregation on the relationship between least-cost and resistance distance. One thousand landscapes were simulated with varying degrees of spatial autocorrelation and least-cost and resistance distance were calculated between 15 sites. The Spearman’s rank correlation (*p*) between these methods was then calculated. These landscapes were then, in parallel, spatially and thematically aggregated by a random factor between 2 and 20. Pairwise distances were once again calculated and subsequently the rank correlations. We then deducted these subsequent rank correlations from the baseline correlations. Thus, |Δ*p*| is the absolute change in rank correlation between methods after aggregation. The degree of spatial aggregation is simply the spatial aggregation factor. The degree of thematic aggregation is ‘22—the number of discrete classes’. This means that if cost values were aggregated into 2 discrete classes the degree of thematic aggregation was 20. This figure illustrates that both spatial and thematic aggregation increase the difference between the rankings of the distances of both methods. Consequently, these methods increasingly disagree as aggregation increases.

**Fig 7:**
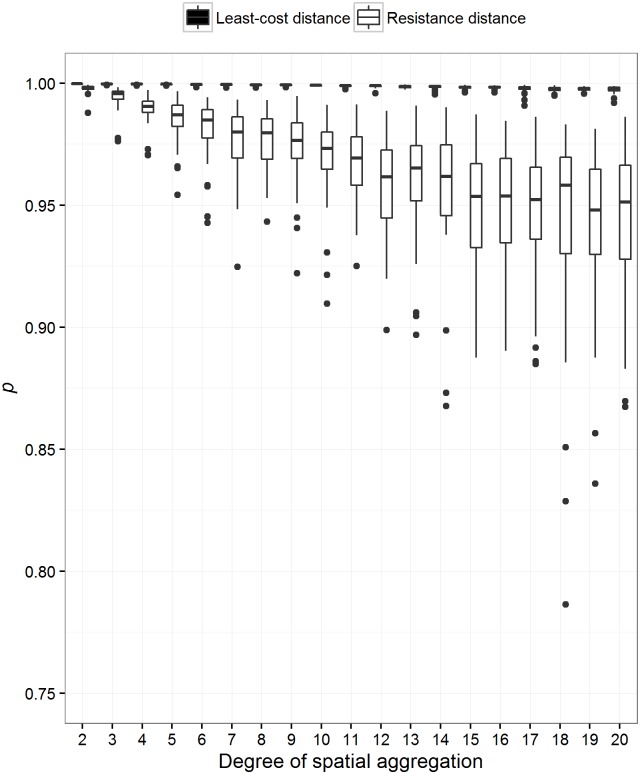
The effect of spatial aggregation on each method. The effect of spatial aggregation on least-cost and resistance distance. One thousand landscapes were simulated with varying degrees of spatial autocorrelation. Least-cost and resistance distance between 15 sites was then calculated for all landscapes. These landscapes were then spatially aggregated by a random factor between 2 and 20. Pairwise distances were once again calculated. We then calculated the Spearman’s rank correlation between each method before and after aggregation. The degree of spatial aggregation is simply the spatial aggregation factor. This figure illustrates the effect of spatial aggregation on either method. Least-cost distance is not affected by spatial aggregation, but the effect of aggregation on resistance distance monotonically increases with the degree of spatial aggregation.

**Fig 8:**
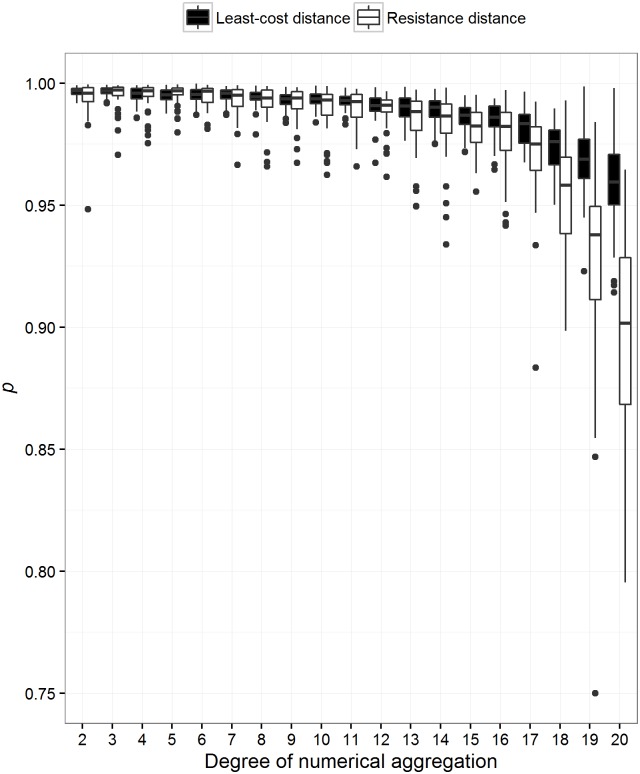
The effect of thematic aggregation on the relationship between least-cost and resistance distance. One thousand landscapes were simulated with varying degrees of spatial autocorrelation. Least-cost and resistance distance between 15 sites was then calculated for all landscapes. The cost values on these landscapes were then aggregated within discrete categorizes. The number of categorizes ranged from 2 to 20 discrete cost values. A cost surface with 20 discrete cost values would represent a land use land cover map and 2 discrete cost values would represent a binary surface. The degree of data aggregation is ‘22—the number of discrete classes’. This means that if cost values were aggregated into 2 discrete classes the degree of thematic aggregation was 20. Pairwise distances were once again calculated. We then calculated the Spearman’s rank correlation between each method before and after aggregation. This figure illustrates that thematic aggregation decreases the agreement between the rankings of the distances. The effect is more apparent for resistance distance compared to least-cost distance.

Both spatial and thematic aggregation decreased the rank correlation between least-cost and resistance distance (Fig 6). However, spatial aggregation had a greater impact on the rank correlation between the two methods. In addition, we tested whether the methods themselves varied independently due to both types of aggregation. We found that spatial aggregation did not affect least-cost distance, but did affect resistance distance (Fig 7). We also found that thematic aggregation affected both methods, but resistance distance to a lesser degree than least-cost distance (Fig 8). Generally, resistance distance was more sensitive to both types of aggregation.

## Discussion

We found that least-cost and resistance distance did not have a direct linear response, but rather, were curvilinear. This was not surprising, as redundancy should scale with area, which is a square. We could linearize the relationship between these measures by using a square-root transformation of least-cost distance or a squared transformation of resistance distance (S1 and S2 Figs):

$$redundancy\prime \;\sim \;\frac{\sqrt{least-cost\;distance}}{\hat{R}}\;\sim \;\frac{least-cost\;distance}{{\hat{R}}^{2}}$$ (2)

where redundancy’ is the linearized version of redundancy after transforming least-cost or resistance distance. In any case, these methods produce somewhat the same rank order (S3 Fig), but there are many cases where their association is quite low (where *p* < 0.7). We can interpret S3 Fig as a probability distribution and infer that in 50% of landscapes the rank correlation between least-cost and resistance distance is < 0.72. This means, 50% of the time the methods have poor agreement in the rankings of their distances. Depending on the type of analysis these differences could be quite important (e.g., [6,19]). Researchers should not use one method as an alternative to the other, since they are not linearly related (Fig 2) and do not agree 50% of the time. They represent difference concepts and additionally function on different spatial and thematic scales.

We also found that commute-time is very similar to resistance distance (S4 Fig), which may be of interest for researchers that use the R statistical language. The commute-time function in the ‘gdistance’ package in R can be used as an alternative to estimating resistance distance in Circuitscape.

Both least-cost and resistance distances estimates responded much differently to the Euclidean distance between focal points, when measured on a log-log scale. They both have linear relationship with Euclidean distance, but the rate of increase of least-cost distance was ~500 times higher than resistance distance. Hence, least-cost distance is more sensitive to changes in Euclidean space when compared to resistance distance. Seeing as Euclidean distance alone explains ~71% of the variance in least-cost distance we could easily approximate least-cost distances on a landscape by simply knowing a few pairwise measures; we could estimate least-cost distance from the Euclidean distance without using Dijkstra’s shortest path algorithm [49].

We had previously thought that spatial autocorrelation would play an important role in explaining the relationship between these methods. However, we did not find any evidence to support this idea (S5 and S6 Figs). This suggests that spatial autocorrelation is not an important consideration when choosing a method for estimating effective distance.

Both metrics also responded differently to the number of pixels representing a landscape (Fig 4). This relationship is quite similar to the Euclidean distance (Fig 3), but in this case the trends are on a log-log scale. Once again, resistance distance had a much smaller rate of change with the number of pixels compared to least-cost distance. Most of the variance in least-cost distance was explained by the number of pixels representing the landscape. If we added the log-transformed Euclidean distance as an explanatory variable, the amount of total variance explained increased to 87.4% for least-cost distance and only 30.6% for resistance distance. This shows that least-cost distance is sensitive to the Euclidean distance and the number of pixels representing a landscape. This is not true to the same extent for resistance distance. Also, we found that there is a gradual trend between the number of pixels representing a landscape and the agreement between the rank orders of both methods (Fig 5). We suggest that there is a baseline correlation between these methods that is a function of the number of pixels. In other words, on landscapes represented by more than 10 million pixels we would expect higher rank correlation compared to a landscape represented by 1 million pixels. This suggests that it should be rare to have a high rank correlation between these methods using data represented by relatively few pixels. It is therefore not surprising that Avon and Bergès [19] found different results when comparing the two methods. Their landscapes were of 7,090 km^2^ and 31,700 km^2^ and their spatial data resolution was 100 m. The number of pixels representing these landscapes was about 0.79 and 3.17 million pixels. Therefore, the baseline Spearman’s rank correlation between these methods would have been between 0.70 and 0.73.

Finally, aggregation is quite common for these types of analyses (e.g., [6,39]). Data manipulation eventually affects the relationship between the methods (Fig 6). Spatial aggregation affects the relationship the most and is a common approach used to reduce processing time and virtual memory usage. For landscapes that are represented by 1 million pixels, spatial aggregation can change the relationship between least-cost and resistance distance on average by 0.11 *p* units, no matter the degree of spatial aggregation. In many cases, data of much finer resolution are represented by many more pixels, consequently aggregation will affect these methods more. It was previously common knowledge that spatial aggregation did not affect resistance distance much, but our results suggest that resistance distance is more sensitive than least-cost distance (Figs 7 and 8). In fact, McRae et al. [33] noted that the pairwise resistance distance between focal nodes from a finer scale habitat map at a resolution of 1000 x 1000 pixels compared to its coarser version of 100 x 100 were highly correlated (R^2^ = 0.963; [33]). However, we found that spatial aggregation by a factor ranging between 2 to 20 produced on average an R^2^ of 0.935 (We squared Spearman’s rank correlation). However, we did not test spatial aggregation of a factor of 100, such as McRae et al [33]. We did find that a 20-fold aggregation produced a correlation (R^2^) between resistance distance of 0.89 (Fig 7). We do suspect an asymptotic response of this correlation.

## Conclusions

In summary, least-cost and resistance distance are not linearly related unless a transformation is applied to either metric. The least-cost distance is partly a function of the number of pixels representing the landscape and the Euclidean distance between focal points. Resistance distance is less sensitive to these factors. Spatial autocorrelation does not affect either method or their relationship. The agreement between these methods is affected by the number of pixels representing the landscape and aggregation. The former is explained by the fact that resistance distance is more sensitive to aggregation than least-cost distance. Consequently, data and data manipulations may govern the differences between these methods and their independent outcome, but not the actually landscape entity being studied. Our findings are relevant for users who wish to evaluate landscape connectivity in a variety of contexts [14], [15], [52]. Researchers should investigate how closely both methods match the biology, behaviour, and ecology of their focal entity. Whether studying gene flow or movement, the biological meaning of least-cost or resistance distance should be appropriate.

## Supporting information

S1 Fig The relationship between square-root of least-cost and resistance distance. Square-root transformation of least-cost distance in relation with resistance distance (See Fig 2). The relationship between least-cost and resistance distance for a subset of 5,000 random pairs sampled from 1000 different simulated landscapes. We had to randomly subset the data to make the figures more visually appealing, since there are 105,000 actual points. Landscape size (number of pixels) is held constant at 1,000,000.
(TIFF)

S2 Fig The relationship between least-cost and resistance distance squared. Least-cost distance in relation with resistance distance squared (See Figs 2 & 3). The relationship between least-cost and resistance distance for a subset of 5,000 random pairs sampled from 1000 different simulated landscapes. We had to randomly subset the data to make the figures more visually appealing, since there are 105,000 actual points. Landscape size (number of pixels) is held constant at 1,000,000.
(TIFF)

S3 Fig The agreement between the rank order of both methods. The distribution of the Spearman’s rank correlation between least-cost and resistance distance for 15 randomly place pairwise focal points on 1000 generated landscapes (μ = 0.720, σ = 0.101, range: 0.246–0.929). Landscape size (number of pixels) is held constant at 1,000,000.
(TIFF)

S4 Fig The relationship between resistance distance and commute-time distance. The relationship between resistance distance from Circuitscape [2] and commute-time from the R package gdistance [48] for a subset of 5,000 random pairs sampled from 1000 different simulated landscapes. The average Spearman’s rank correlation between resistance distance and commute-time for pairwise measure between 15 randomly placed focal points on 1000 generated landscapes was 0.99 (σ = 0.00005). Landscape size (number of pixels) is held constant at 1,000,000.
(TIFF)

S5 Fig The effect of spatial autocorrelation. The relationship between least-cost and resistance distance and the degree of spatial autocorrelation for a subset of 5,000 random pairs sampled from 1000 different simulated landscapes. The y-axis in this graph is on a logarithmic scale for comparison purposes only. Landscape size (number of pixels) is held constant at 1,000,000.
(TIFF)

S6 Fig Spatial autocorrelation and rank order agreement. The association between least-cost and resistance distance for pairwise measurements of 15 pairwise focal points on 1000 generated landscapes with varying degrees of spatial autocorrelation. The range of spatial autocorrelation of a landscape does not affect the association between least-cost and resistance distance. Landscape size (number of pixels) is held constant at 1,000,000.
(TIFF)

S1 File Rscript.zip. Compressed R scripts used to perform all the analyses mentioned in the “Materials and Methods” section.
(ZIP)
