# Supplementary figures and images for: The relationship between least-cost and resistance distance
Source: PLoS One. 2017 Mar 28;12(3):e0174212. doi: 10.1371/journal.pone.0174212 (PMC5369686; doi:10.1371/journal.pone.0174212)

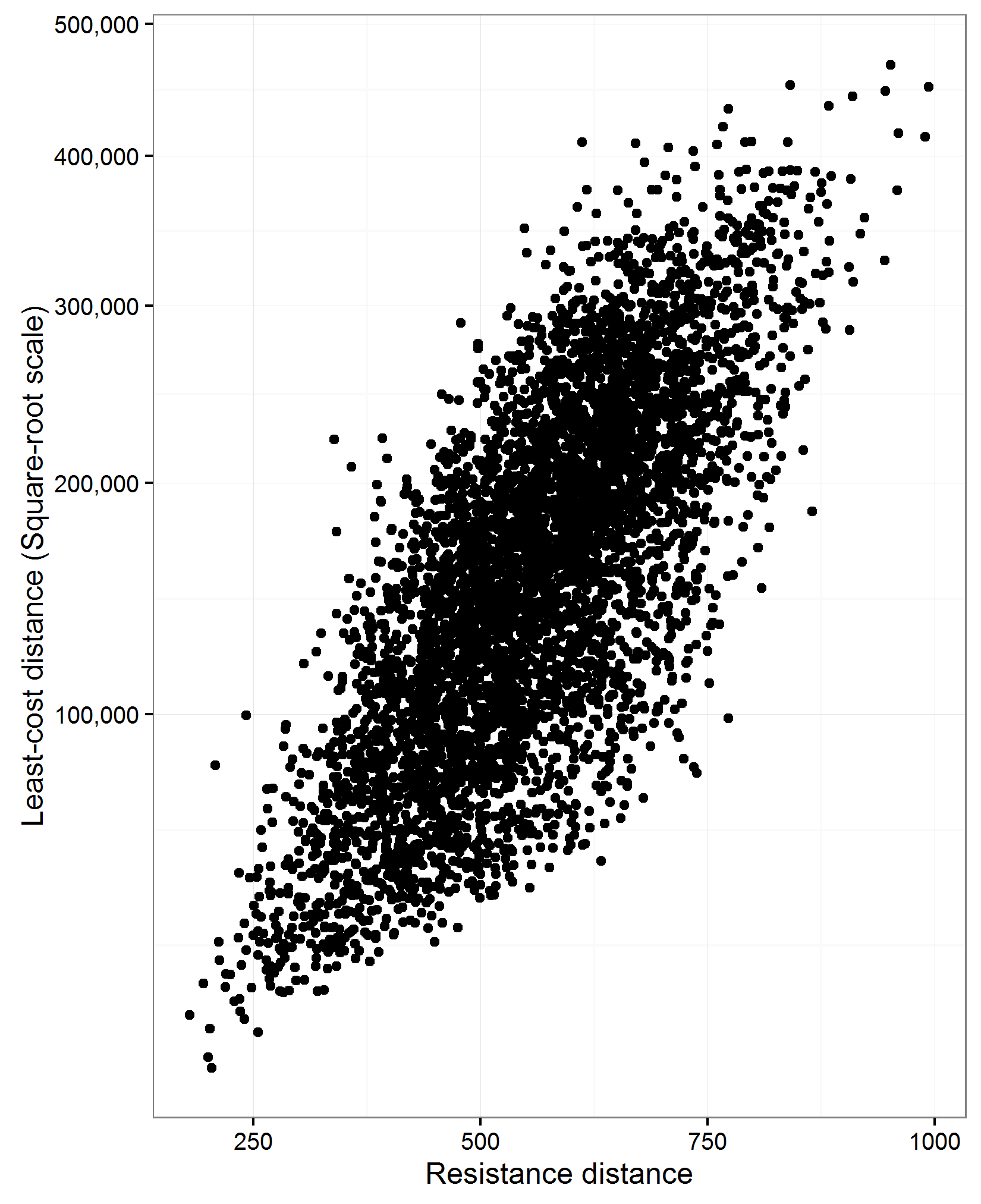

Supplement: S1 Fig — Square-root transformation of least-cost distance in relation with resistance distance (See Fig 2). The relationship between least-cost and resistance distance for a subset of 5,000 random pairs sampled from 1000 different simulated landscapes. We had to randomly subset the data to make the figures more visually appealing, since there are 105,000 actual points. Landscape size (number of pixels) is held constant at 1,000,000. (TIFF) [file pone.0174212.s001.tiff]

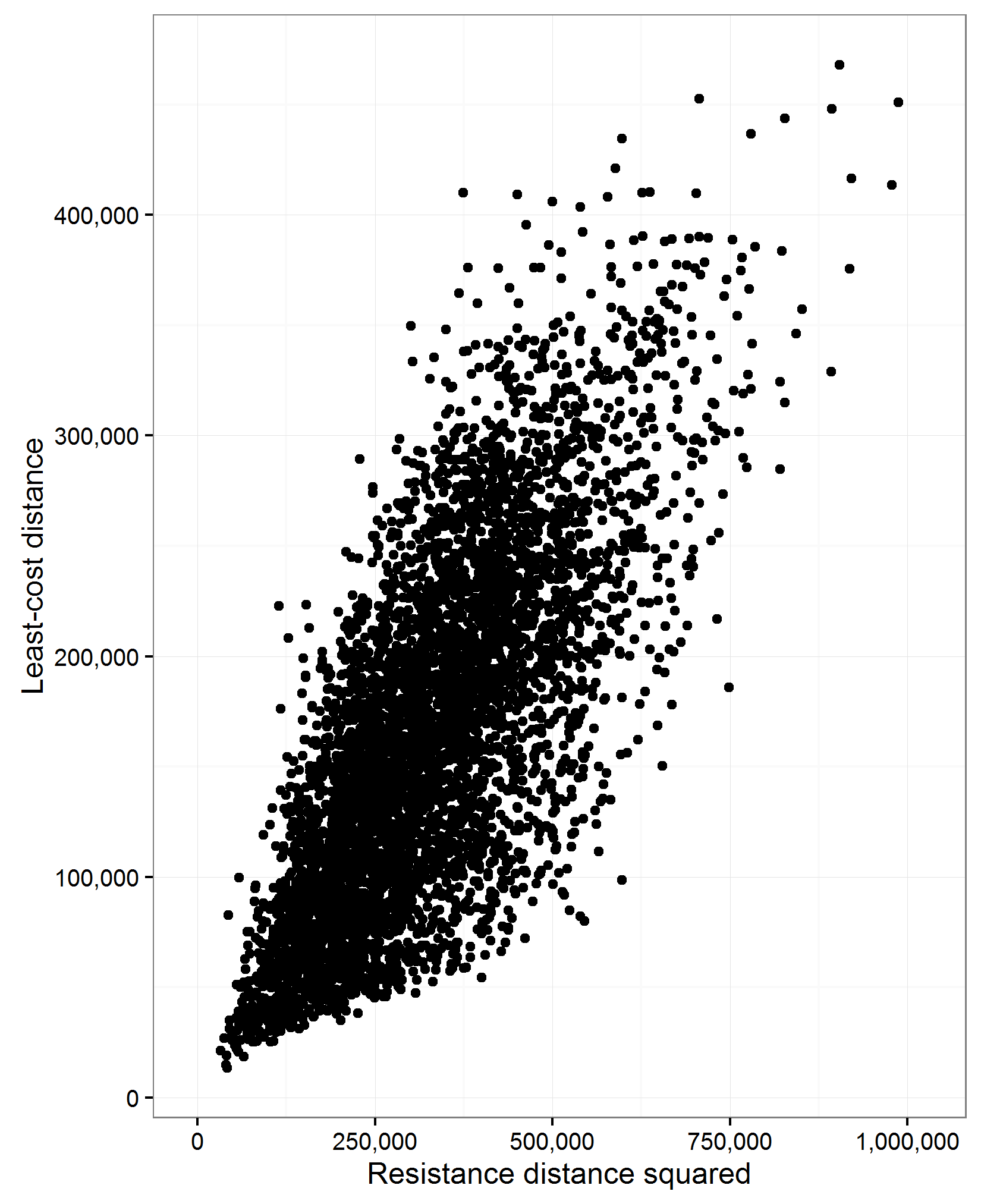

Supplement: S2 Fig — Least-cost distance in relation with resistance distance squared (See Figs 2 & 3). The relationship between least-cost and resistance distance for a subset of 5,000 random pairs sampled from 1000 different simulated landscapes. We had to randomly subset the data to make the figures more visually appealing, since there are 105,000 actual points. Landscape size (number of pixels) is held constant at 1,000,000. (TIFF) [file pone.0174212.s002.tiff]

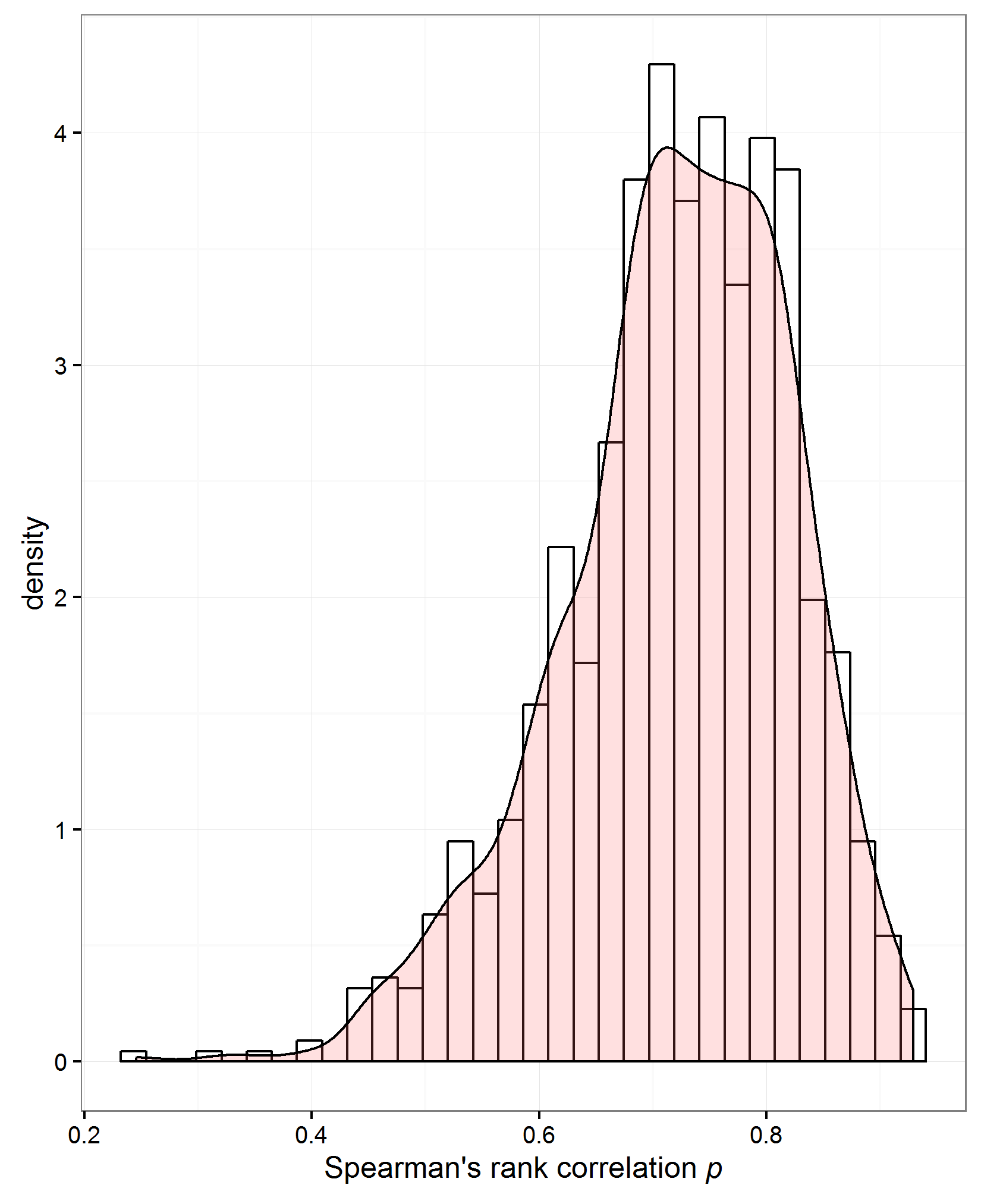

Supplement: S3 Fig — The distribution of the Spearman’s rank correlation between least-cost and resistance distance for 15 randomly place pairwise focal points on 1000 generated landscapes (μ = 0.720, σ = 0.101, range: 0.246–0.929). Landscape size (number of pixels) is held constant at 1,000,000. (TIFF) [file pone.0174212.s003.tiff]

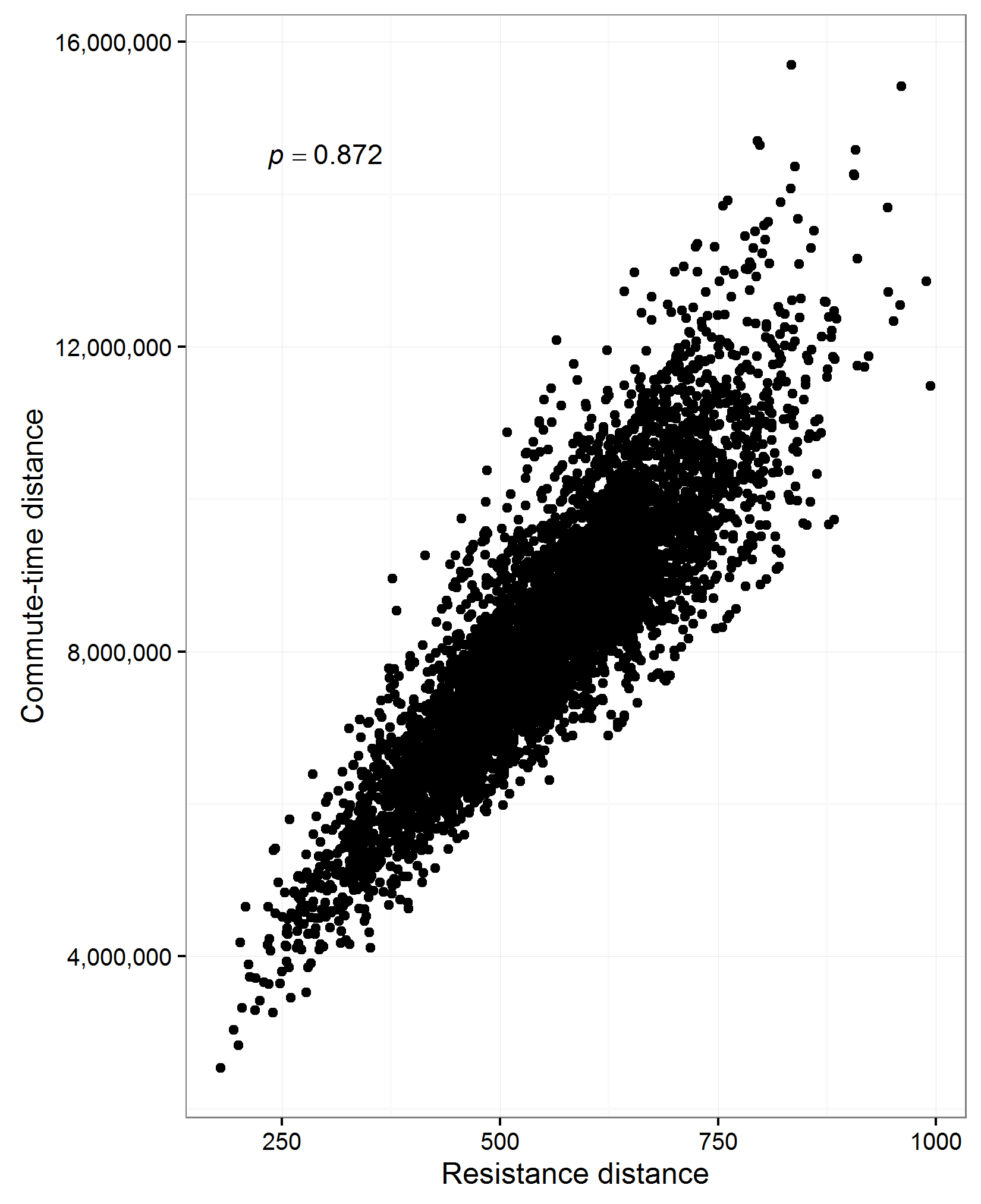

Supplement: S4 Fig — The relationship between resistance distance from Circuitscape [2] and commute-time from the R package gdistance [48] for a subset of 5,000 random pairs sampled from 1000 different simulated landscapes. The average Spearman’s rank correlation between resistance distance and commute-time for pairwise measure between 15 randomly placed focal points on 1000 generated landscapes was 0.99 (σ = 0.00005). Landscape size (number of pixels) is held constant at 1,000,000. (TIFF) [file pone.0174212.s004.tiff]

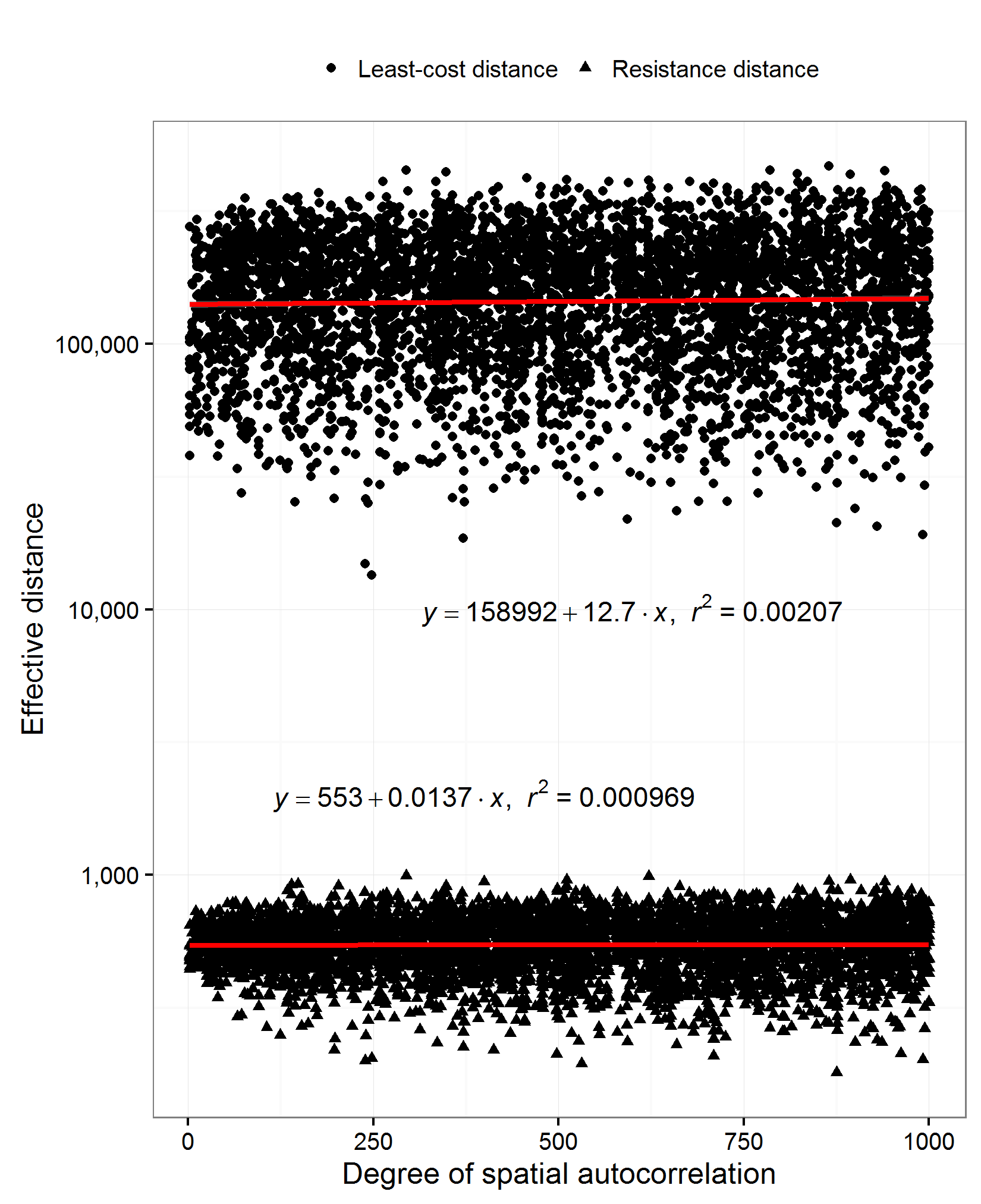

Supplement: S5 Fig — The relationship between least-cost and resistance distance and the degree of spatial autocorrelation for a subset of 5,000 random pairs sampled from 1000 different simulated landscapes. The y-axis in this graph is on a logarithmic scale for comparison purposes only. Landscape size (number of pixels) is held constant at 1,000,000. (TIFF) [file pone.0174212.s005.tiff]

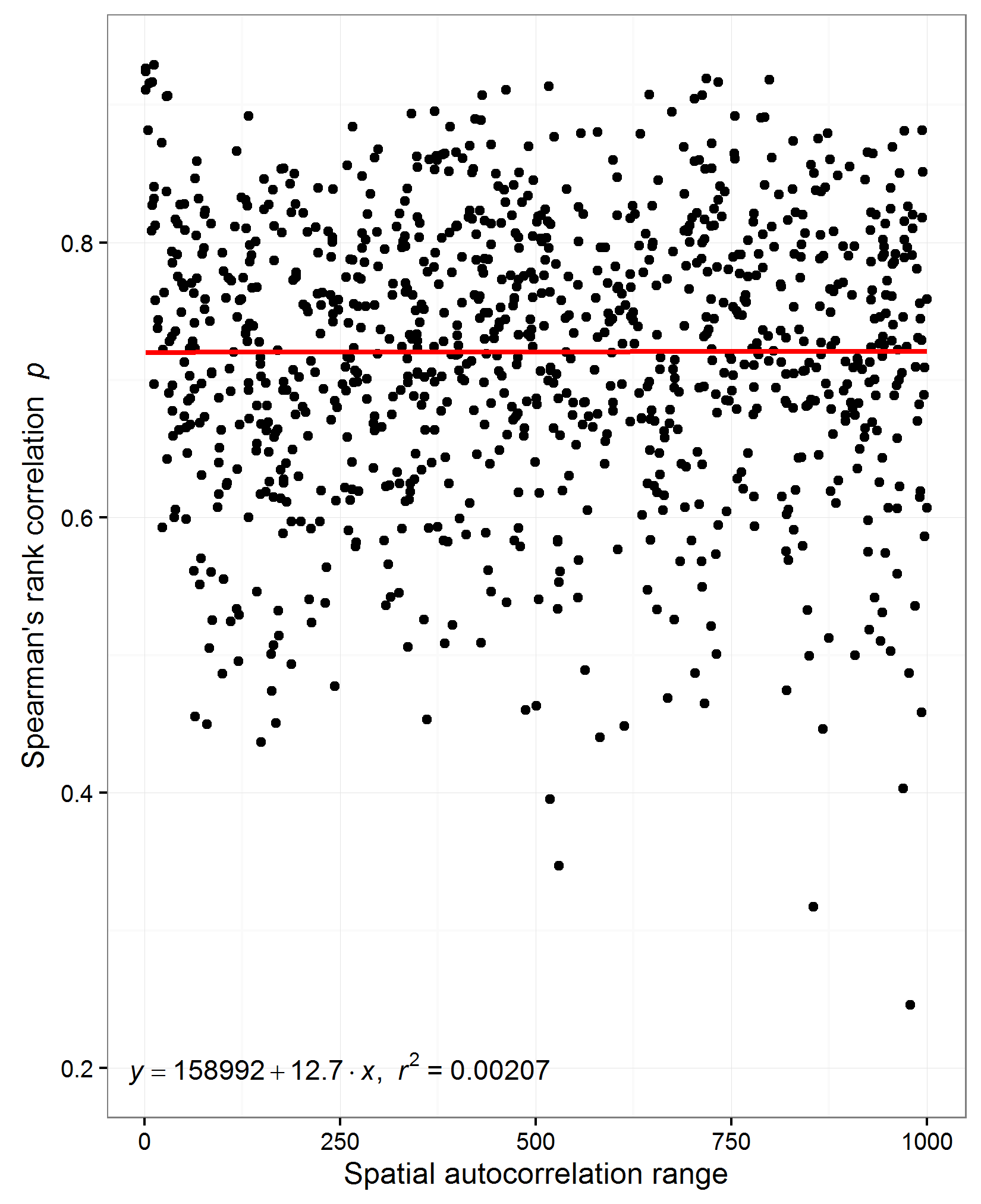

Supplement: S6 Fig — The association between least-cost and resistance distance for pairwise measurements of 15 pairwise focal points on 1000 generated landscapes with varying degrees of spatial autocorrelation. The range of spatial autocorrelation of a landscape does not affect the association between least-cost and resistance distance. Landscape size (number of pixels) is held constant at 1,000,000. (TIFF) [file pone.0174212.s006.tiff]
